# Effect of Tomato Pomace Addition on Chemical, Technological, Nutritional, and Sensorial Properties of Cream Crackers

**DOI:** 10.3390/antiox11112087

**Published:** 2022-10-22

**Authors:** Gjore Nakov, Andrea Brandolini, Lorenzo Estivi, Katia Bertuglia, Nastia Ivanova, Marko Jukić, Daliborka Koceva Komlenić, Jasmina Lukinac, Alyssa Hidalgo

**Affiliations:** 1 College of Sliven, Technical University of Sofia, 59 Bourgasko Shaussee Blvd., 8800 Sliven, Bulgaria; 2 Consiglio per la Ricerca in Agricoltura e L’analisi Dell’Economia Agraria—Centro di Ricerca Zootecnia e Acquacoltura (CREA-ZA), Via Forlani 3, 26866 Sant’Angelo Lodigiano, Italy; 3 Department of Food, Environmental and Nutritional Sciences (DeFENS), Università degli Studi di Milano, Via Celoria 2, 20133 Milano, Italy; 4 Faculty of Food Technology Osijek, Josip Juraj Strossmayer University of Osijek, Str. Franje Kuhača 18, HR-31000 Osijek, Croatia

**Keywords:** carotenoids, antioxidant capacity, by-products, flavonoids, fibre, HPLC, phenolic acids, tocols

## Abstract

The aim of this research was to determine the influence of tomato pomace (TP) addition on the chemical, nutritional, and technological characteristics of cream crackers made from wheat flour and 4%, 6%, 8%, and 10% TP. The TP-enriched cream crackers showed progressively increasing ash (from 0.69 of the control to 1.22 g/100 g dry matter of the 10% TP sample), fat (from 11.39 to 13.04 g/100 g), protein (from 13.53 to 15.60 g/100 g), total dietary fibre (from 4.08 to 7.80), carotenoids (from 0.55 to 8.56 mg/kg), tocols (from 57.59 to 71.63 mg/kg), free phenolic acids (from 100.08 to 277.37 mg/kg), free flavonoids (from 0.0 to 45.28 mg/kg), bound flavonoids (from 0.0 to 27.71 mg/kg), and fatty acids contents, antioxidant activity and dough viscosity. The colour coordinates increased via augmenting the amounts of TP. Thickness, volume, and specific volume decreased gradually with increasing TP; the enrichment reduced cracker hardness from 65.42 N (control) to 26.28 N (crackers with 10% TP), while the snapping force rose. Cream crackers with 8% TP showed the best sensory quality. Tomato pomace addition improves the nutritional quality of foods; furthermore, its recycling will help to solve the problems linked to the disposal of this industry waste.

## 1. Introduction

By-product management during fruit and vegetable processing is one of the biggest problems for the agro-food industry, because often large quantities of leftover pomace are thrown directly in landfills, causing serious environmental problems [1]. In a circular economy, waste materials are advantageously recycled as new raw materials. Presently, pomace is used as soil fertilizer [2] or as animal food [3], but it could be better employed as raw material to produce new functional products for human nutrition.

Tomato (*Lycopersicon esculentum* L.) is one of the most consumed fruits/vegetables in the world, with a 2020 annual production of 187 million tons [4]. Tomatoes are eaten fresh or are processed into tomato juice, tomato paste, etc. The waste, commonly called tomato pomace (TP), contains peels, seeds, and small pulp leftovers [5], and is around 4 million tons a year [6]. The recycling of this waste may be difficult because of rapid microbial de-composition; on the other hand, recycling is environmentally important and economically beneficial because TP contains important biologically active substances. The nutritional value of TP largely depends on its composition. In fact, tomatoes contain antioxidant compounds, such as tocols and carotenoids [7], phenolic compounds, vitamins C and E [8,9], as well as minerals (potassium, phosphorus, magnesium, calcium, and sodium) [10]; the peels have 2.5 times more lycopene than seeds and pulp [11], while the seeds are rich in protein, polyphenols, fibre, and lipids (mainly linoleic, oleic, and palmitic fatty acids) [12,13]. Due to the excellent nutritional components, after drying and grinding, TP can be used as raw material for the preparation of new products with nutritionally enhanced characteristics. So far, several studies have been conducted on the effect of tomato supplementation on soda crackers [14], bread and muffins [10], extruded barley snacks [15], biscuits [16], and cakes [17], but to the best of our knowledge, no information about the influence of tomato pomace on the antioxidant fate of bakery products, in particular cream crackers, is currently available. Hence, this study aims to evaluate the effects of the addition of increasing tomato pomace quantities (0%, 4%, 6%, 8%, and 10%) on the antioxidant properties (profile of carotenoids, tocols, free phenolics and bound phenolics, and antioxidant capacity) and on the physical, chemical, technological, and sensory characteristics of cream crackers.

## 2. Materials and Methods

### 2.1. Materials

T-550 commercial all-purpose wheat flour (Tena-Žito Ltd., Đakovo, Croatia) was used to produce the cream crackers. The tomato pomace (TP) was produced from ripe tomatoes grown in 2019 in the Razgrad region, Bulgaria. After removing the pulp, TP was dried for 48 h at 45 °C in a UFE 500 oven (Memmert GmbH, Schwabach, Germany), then ground (powder size < 0.5 mm) with an IKA MF10 grinder (IKA^®^-Werke GmbH & Co. KG, Staufen, Germany) and stored at −18 °C. The margarine (Zvijezda Ltd., Zagreb, Croatia), with a total fat content of 70%, contained vegetable fats (palm oil), water, vegetable oils (sunflower, rapeseed), iodised salt 0.4%, emulsifier (E 471), preservative (E 200), acidity regulator (citric acid), flavouring, and colouring (β-carotene). The other ingredients were bought from local stores in Osijek, Croatia.

### 2.2. Methods

#### 2.2.1. Cream Crackers Preparation

The cream crackers were prepared according to Nakov and Ivanova [18], with minor changes. In short, to a dough made by blending wheat flour (250 g), margarine (37.5 g), salt (3.75 g), and sugar (3.125 g) with a MMC700W mixer (Gorenje, Velenje, Slovenia) for 3 min were added 70 mL water and a yeast suspension prepared with 6.25 g fresh yeast and 30 mL water at 35 °C. The resulting dough was mixed for 2 min at high speed and then rested for 24 h at 30 °C in a fermentation chamber. The enriched crackers were made by substituting wheat flour with 4%, 6%, 8%, and 10% TP, percentages chosen after thorough research of the scientific literature. The crackers without TP added (0%) served as a control sample. The fat dough was prepared by mixing for 2 min 62.5 g flour, 0.94 g salt, 1 g baking powder, and 40 g margarine with the above-mentioned MMC700W mixer.

The leavened dough was reduced to 6 mm thickness with a dough sheeter (Professional LMP500 laboratory laminator, Electrolux, Stockholm, Sweden). A 1 mm layer of fat dough was placed over the cracker dough, and the resulting double layer was folded positioning the fat layer inside two dough layers, and then it was laminated and folded again. After resting for 30 min at 4 °C in a polyethylene bag, four further laminations were performed, interspersed with three additional dough rests. Finally, the cream crackers were formed with a manual cutter, their surfaces were pierced to facilitate quick CO_2_ release, and they were baked at 240 °C for 7 min in a convection oven (Wiesheu Minimat Zibo, Wiesheu GmbH, Großbottwar, Germany). Five types of crackers were prepared, i.e., control crackers (100% wheat flour), and crackers enriched with 4%, 6%, 8%, and 10% TP. Two 40-cracker sets were produced from each flour/enriched mix.

#### 2.2.2. Pasting Properties

The pasting properties of the wheat flour and the TP-enriched mixtures were tested using a Micro ViscoAmylo-Graph (Brabender OHG, Duisburg, Germany). The following parameters were determined: peak viscosity, breakdown, setback and final viscosity (in Brabenber Units, BU), and pasting temperature (in °C). The measurements were performed in duplicate.

#### 2.2.3. Physical Characteristics

**Figure 1:**
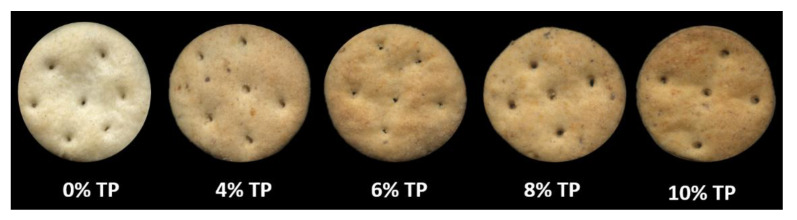
Cream crackers enriched with 0%, 4%, 6%, 8%, and 10% tomato pomace (TP).

Mean thickness and width (mm) were measured on five crackers. The colour of dough and crackers in the CIE L*, a*, b* system was scored with a Chroma Meter CR-400 colorimeter (Konica Minolta, Tokyo, Japan) with the use of illuminant D65 on two sets of five random crackers. All data were presented as mean values of at least five replicates from each batch. The volume (cm^3^) was determined on three samples using a Volscan Profiler (Stable Micro Systems Ltd., Godalming, UK). The spread factor was calculated as width/thickness multiplied by 10. The specific volume was calculated as volume divided by weight of the same cracker. The textural properties of the crackers (hardness and snapping force) were measured using a TA-XT2 Plus texture analyser (Stable Micro System Ltd., Godalming, UK). Images of cream crackers with different quantities of tomato pomace are shown in Figure 1.

#### 2.2.4. Chemical Characteristics

Moisture and ash contents of wheat flour, TP, and crackers were determined according to methods ISO 665:2020 [19] and ISO 5984:2002 [20], respectively; protein content was assessed by the modified Lowry method, as described in Mæhre et al. [21]; lipid concentration was tested according to Soxhlet method ISO 136 [22]; and fibre content (soluble, insoluble, and total) was assessed as in method 32–07.01 [23]. Total carbohydrate content was computed by subtracting protein, lipid, and ash from the total. The results are reported as g/100 g dry matter (DM).

Identification of fatty acids in tomato pomace was performed using a Shimadzu-2010 gas chromatograph (Kyoto, Japan). The assay was performed with a CP7420 capillary column (100 × 0.25 mm, i.d. 0.2 μm, Varian Inc., Palo Alto, CA, USA) with carrier gas (hydrogen) and make-up gas (nitrogen). A gas chromatographic oven with five-step temperature program was used. All the analyses were performed in duplicate.

#### 2.2.5. Tocols and Carotenoids

The analyses of tocols, trans-lutein, and trans-zeaxanthin were scored by normal phase HPLC, while lycopene and carotenoid isomers were analysed by reverse phase HPLC.

The tocol and carotenoid extracts were obtained from all the samples after saponification [24]. The NP-HPLC analysis was performed as described by Hidalgo and Brandolini [25]. The operating conditions for tocol identification were: Adamas^®^ Silica column 250 × 4.6 mm, 5 μm, and guard cartridge 10 × 4.6 mm, 5 μm (Sepachrom SRL, Rho, Italy); mobile phase, hexane:ethyl acetate:acetic acid (97.3:1.8:0.9, *v*/*v*/*v*); flow rate, 1.6 mL/min; pump L-2130 Elite LaChrom (VWR, Hitachi, Japan); fluorimetric detector Jasco 821 FP Intelligent Spectrofluorometer (Tokyo, Japan) at excitation-emission wavelengths of 290 nm and 330 nm, respectively; connected to a computer with the software Empower 2 (Waters Chromatography Division, Millipore, Milford) through the Waters e-SAT/IN module. Peaks were quantified using α-tocopherol, β-tocopherol, γ-tocopherol, and δ-tocopherol as external standards. The tocotrienols were quantified using the standard curves of their corresponding tocopherol. The operating conditions for lutein and zeaxanthin detection [26] were: Adamas^®^ Silica column 250 × 4.6 mm, 5 μm, and guard cartridge 10 × 4.6 mm, 5 μm (Sepachrom SRL, Rho, Italy); column oven at 20 °C L-2300 Elite LaChrom (VWR, Hitachi, Japan); mobile phase, hexane:isopropyl alcohol (5%); flow rate, 1.5 mL/min; pump L-2130 Elite LaChrom (Hitachi, Tokyo, Japan). Carotenoids were detected at 445 nm by a PhotoDiode Array Detector (Waters, Milford, MA, USA) set in the range of 200–650 nm. The HPLC system was controlled by the software Empower 2. The lycopene and carotenoid isomer quantification was carried out by reverse phase HPLC; the samples were prepared as described by Hidalgo et al. [27]. The carotenoid chromatographic analysis was carried out following Gupta et al. [28]. Exactly 20 μL volume of filtrate was tested under the following operating conditions: L-2130 Elite LaChrom pump (VWR, Hitachi, Japan), L-2300 Elite LaChrom column oven (VWR, Hitachi, Japan), diode Array Detector L2450 Elite LaChrom (Merck, Hitachi, Japan), at wavelength 445 nm, with EZChrom Client/Server software version 3.1.7. The analysis was carried out using an Adamas C30 column 5 μm–250 mm × 4.6 mm—HPLC Column (Sepachrom Srl, Rho, Italy), an Adamas pre-column thermostated at 20 °C, using mobile phases consisting of (A) methanol/water (98: 2, *v*/*v*), (B) methanol/water (95: 5, *v*/*v*), and (C) tert-methyl butyl ether. The gradient elution was 80% A, 20% C at 0 min, followed by a linear gradient at 60% A, 40% C at 2.0 min at a flow rate of 1.4 mL/min, at 2.01 min flow rate was changed to 1.00 mL/min with gradient changing to 60% B, 40% C followed by a linear gradient at 0% B, 100% C within 12 min and returning to initial conditions within 13 min. A rebalancing (7 min) was performed at initial concentrations of 80% A, 20% C. The column temperature was maintained at 20 °C. Elution peaks were monitored in a range of 250 to 700 nm using PDA. The quantification of the peaks was performed with the external standard method by preparing calibration curves of lycopene and β-carotene for all the other compounds. The analyses were carried out in duplicate and the results expressed in mg/kg dry matter (DM).

#### 2.2.6. Free and Bound Phenolic Compounds

The extraction of the soluble free and insoluble bound phenolic compounds was carried out according to the procedure reported by Brandolini et al. [29]. Approximately 1 g of flour was weighed into capped centrifuge tubes, and 15 mL of a methanol solution (80%) was added to each tube. After vortexing with a Mn1Minishaker (Ika, Staufen, Germany) for about 45–60 s, the samples were placed in an ultrasonic bath for 10 min, vortexed again for a few seconds, and centrifuged at 12,000 RPM for 10 min at 8–9 °C with a Centrikon K24 centrifuge (Kontron Instruments, Bletchley, UK). The supernatants, which contain the free phenolics, were recovered in a 250 mL flask, covered with foil to protect the content from light. The extraction was repeated two more times. The three extracts were merged, evaporated under vacuum using a Laborota 4000 rotavapor (Heidolph Instruments GmbH & Co. KG, Schwabach, Germany) for 40 min at 35 °C and completely dried by nitrogen flow for 1 min. The dry samples were resuspended with 2 mL of methanol:chromatographic water (8:2 *v*/*v*), vortexed, and filtered on a 0.45 μm PTFE membrane (Diana Beck Scientific, Angera, Italy).

The solid residues, which contained the insoluble bound phenols, were digested with 15 mL of 4M NaOH under nitrogen for 4 h at room temperature and constant agitation, brought to a pH of 1.5–2 with 6M HCl, and extracted twice with 20 mL of diethyl ether/ethyl acetate (1:1, *v*/*v*). After centrifugation (11,178 g, 10 min, 8 °C), the supernatants were clarified with sodium sulphate, filtered, evaporated as previously outlined, resuspended in 2 mL of methanol:water (1:1 *v*/*v*), and filtered with a 0.22 μm PTFE membrane (Millipore, Carrigtwohill County, Cork, Ireland). All extractions were performed under dim light, and the extraction tubes were wrapped with black paper to avoid sample degradation by photooxidation.

A 20 µL volume of filtrate underwent reverse phase HPLC analysis using a column Adamas^®^ C18-AQ 5 μm 4.60 mm × 250 mm and a precolumn C18 5 μm 4.60 mm × 10.0 mm (Sepachrom SRL, Rho, Italy) thermostated at 30.0 °C; L-2130 pump, L-2300 column oven and L2450 Diode Array Detector Elite LaChrom (Hitachi, Tokyo, Japan). The identity of the compound was confirmed by the congruence of retention times and UV/VIS spectra with those of pure authentic standards. Thirty-three standards were injected; unidentified peaks were quantified using the calibration curve of the compound with a similar absorption spectrum and named as “phenolic derivative” as indicated in Brandolini et al. [29] and according to Dueñas et al. [30], Zalewski et al. [31], and Estivi et al. [32]; additionally, the derivatives of each compound were grouped together. The following calibration curves were constructed using 4–6 concentrations: apigenin 1–20 mg/L (detection limit 1.19 mg/L), diosmin 5.24–104.8 mg/L (0.41 mg/L), catechin 13.9–99.2 mg/L (1.86 mg/L), genistein 27.5–110.0 mg/L (1.52 mg/L), naringenin 2.25–9.0 mg/L (0.15 mg/L), tyrosol 3.93–98.2 mg/L (1.4 mg/L), cinnamic acid 4.05–19.1 mg/L (0.19 mg/L), 4-hydroxybenzoic acid 1.05–70.4 mg/L (0.19 mg/L), p-coumaric acid 0.80–3.50 mg/L (0.04 mg/L), syringic acid 1.03–10.9 mg/L (0.24 mg/L), ferulic acid 2.61–10.4 mg/L (0.04 mg/L), gallic acid 2.2–101.6 mg/L (4.36 mg/L), quercitin 1.2–21.6 mg/L (0.88 mg/L), rutin 19.06–136.11 mg/L (5.14 mg/L), and caffeic acid 8.73–174.6 mg/L (2.55 mg/L). The results are reported as mg/kg DM.

#### 2.2.7. Antioxidant Capacity

The samples were extracted as suggested by Varas Condori et al. [33] with hexane (lipophilic fraction) and 80% methanol (hydrophilic fraction) in succession. Exactly 0.100 ± 0.010 g of sample was weighed in Eppendorf tubes and resuspended with 1 mL hexane solution. The suspension was vortexed with a WIZARD Advanced IR Vortex Mixer (Velp Scientifica Srl, Usmate, Italy) and sonicated (Bandelin Sonorex, Berlin, Germany) for 10 min at 4 °C in the dark; subsequently, the samples were centrifuged (Centrifuge mod. 4224, ALC, Winchester, MA, USA) at 12,000 rpm for 10 min. The supernatant was thus recovered, and the extraction was repeated; the second supernatant was then combined with the previous one. For the hydrophilic fraction, 80% methanol was used on the sediment with a procedure similar to the previous one.

The antioxidant capacity of the lipophilic extracts was tested by the ABTS method [34]. To 150 µL of extract, 3.0 mL of a radical solution consisting of 10 mL of aqueous 7 mM ABTS solution and 170 µL of 140 mM aqueous potassium persulfate solution (absorbance value of 0.70 ± 0.02) were added and subjected to 6 min of reaction at 30 °C in a thermostated bath (EN.CO. Srl, Spinea, Italy). The absorbance of the extracts was measured at 734 nm using plastic cuvettes with a spectrophotometer (JASCO V-650 Spectrophotometer, Tokyo, Japan).

The antioxidant capacity of the hydrophilic fraction was measured by the ABTS method [34], as described above, and also by the FRAP method [35], slightly modified. Exactly 3.0 mL of the FRAP reagent were added to 150 μL of the extracted sample and, after 60 min of reaction at 37 °C in a thermostated bath (Thermo Electron Corporation, EN.CO. Srl, Rodano, Italy), the absorbance was measured at 593 nm using cuvettes with a spectrophotometer (JASCO V-650 Spectrophotometer, Tokyo, Japan). Both analyses were carried out in duplicate, and the antioxidant capacity was expressed in mmol of Trolox equivalent (TE)/kg DM.

#### 2.2.8. Sensory Analysis

The sensory analysis of the five types of cream crackers with different TP contents was performed at the sub-department of Cereal Technology at the Faculty of Food Technology in Osijek, Croatia. Twenty trained people, after giving informed consent according to the guidelines on Ethics and Food Related research defined by the European Union [36], participated in the sensory tests. Appearance, texture, odour, aroma, and taste were scored from 1 to 5, where 1 was extreme dislike and 5 was extreme like; the overall quality was computed as averages of the five traits evaluated.

#### 2.2.9. Statistical Data Analysis

The experimental data were analysed by one-way analysis of variance (ANOVA). When significant differences (*p* ≤ 0.05) were found, Fisher’s least significant difference (LSD) at *p* ≤ 0.05 was computed. The analyses were performed using the Centurion XVI statistical program (Statgraphics Technologies Inc., The Plains, VA, USA). The mean and standard deviation values were calculated using the Office Excel program (Microsoft Corporation, Redmond, WA, USA).

## 3. Results and Discussion

### 3.1. Rheological Properties

Table 1 reports the results of peak viscosity, breakdown, setback, final viscosity, and pasting temperature of the wheat flour and of the TP-enriched mixtures. The ANOVA showed that substituting wheat flour with TP significantly influenced (*p* ≤ 0.05) all these parameters. The progressive increase in final viscosity with the addition of TP may be related to the higher content of dietary fibre (cellulose, hemicellulose, and pectin), which acts as a hydrocolloid [37] and has a higher gelatinization capability. Due to the content of insoluble dietary fibre and protein (mostly coming from the tomato skin), TP can be a component for altering water sorption and regulating the rheological properties of food [38].

Nakov et al. [39] noticed a similar trend in the dough of cakes enriched with grape seed powder and attributed it to the high content of soluble dietary fibre, and/or to the high lipid content of TP, which may interact with other hydrophobic substances (e.g., gluten), thus increasing viscosity. On the other hand, Mironeasa and Gabriela [40] noticed a decrease in final viscosity, attributed to the increase in tomato pomace, a non-starch component, and to the concomitant reduction of wheat flour. Similarly, Sogi et al. [41] analysed the effect of tomato seed meal on the rheological characteristics of dough and noticed that replacing wheat flour with 10%, 20%, and 30% tomato seed meal did not change pasting temperature, but peak viscosity, breakdown, setback, and final viscosity decreased.

### 3.2. Physical Characteristics

Table 2 shows the colour of the dough and of the cream crackers, as well as the physical and textural characteristics of the cream crackers produced without (control) or with TP addition. In general, all these parameters varied according to the TP content of the cream crackers.

The colour of dough and crackers became darker (lower L*) by increasing the TP amount, while a* and b* increased; that is, they became redder and yellower. The same results, due to the rich pigment content of TP, were reached by other authors [10,42].

**Figure 2:**
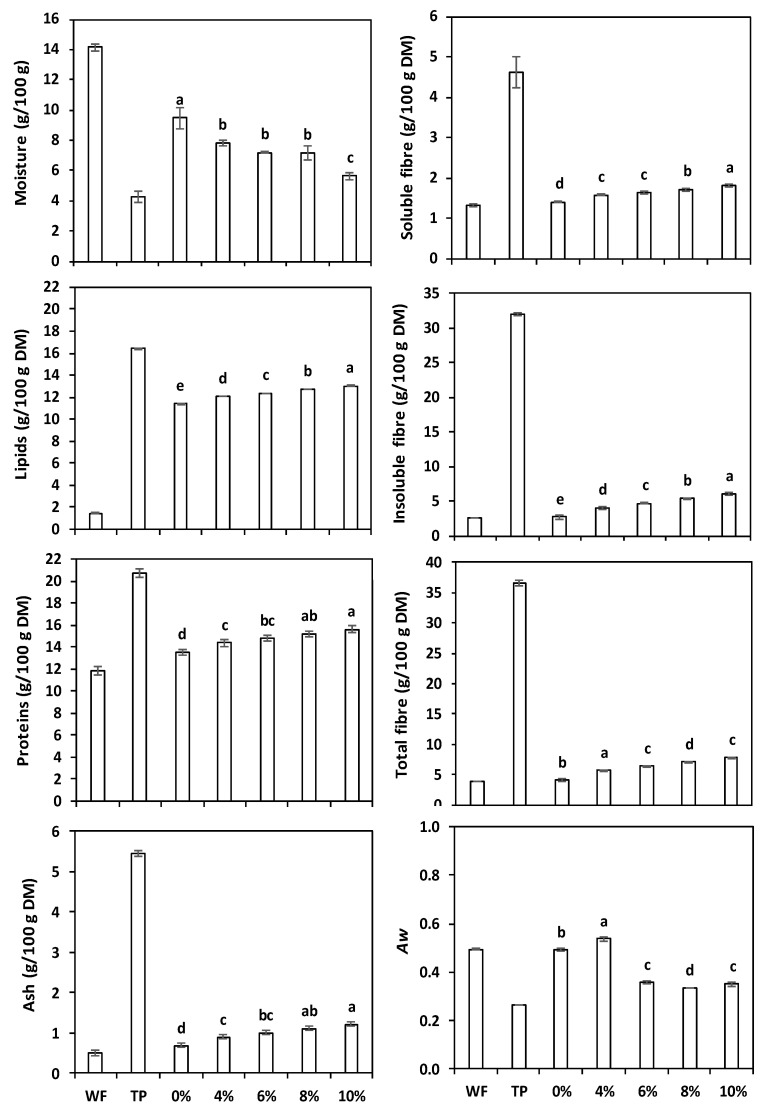
Moisture, composition, and water activity (Aw) of bread wheat flour (WF), tomato pomace (TP), and crackers prepared with increasing TP percentage. The error bars represent the standard deviation; different letters indicate significant differences (*p* < 0.05) among cream crackers.

No significant width differences were detected, whereas the thickness and volume of the 8% and 10% TP crackers were significantly lower (*p* < 0.05), and the spread factor was higher than the control. It has to be stressed, however, that the incorporation of even the lowest TP amount (4%) caused a significant reduction (*p* < 0.05) in volume and specific volume. Bhat and Ahsan [42], Chung [43], and Bhat et al. [44] observed that adding more than 5% TP in biscuits significantly changed the diameter and thickness, probably because the higher TP fibre content diminished gas retention. A volume reduction was expected, because the wheat flour (rich in gluten, responsible for leavened products’ volume) was replaced by the gluten-free TP. Additionally, bakery products’ volume also depends on the ability to produce gases, which are linked to many factors such as growth agents, starch, fermentable sugars, pH, etc., but not to dietary fibre [45]. TP is rich in fibre (Figure 2); therefore, this factor can lead to reduced cream cracker volumes. Nakov et al. [39] noticed that adding grape pomace to cakes led to width, thickness, and volume reductions proportional to the grape pomace content.

TP addition significantly reduced cracker hardness from 65.42 N (control) to 26.28 N (crackers with 10% TP), and increased snapping force (the force required to break the cracker; Jukić et al. [46]) according to TP content. Chung [43] found a decrease in hardness from 60.35 kg/cm^2^ (control cookies) to 38.98 kg/cm^2^ (cookies with 10% TP), while Mehta et al. [10] had similar results in TP-enriched bread and muffins compared to the control samples. Probably, the hydrocolloid nature of TP inhibited the connection between starch chains and reduced starch retrogradation [37].

### 3.3. Chemical Characteristics

The ANOVA (not shown) proved the existence of significant differences (*p* < 0.05) between the control and the crackers with different amounts of TP for all parameters. Figure 1 shows the main chemical characteristics of white wheat flour and tomato pomace, as well as of crackers with and without TP.

The wheat flour moisture was far higher than that of the tomato pomace, as already evidenced by other authors [37,42]. Hence, its reduction with increasing TP percentage was observed, going from 9.46 g/100 g (control) to 5.64 g/100 g (crackers with 10% TP). Moisture reduction in biscuits with different amounts of TP was reported by Chung [43] and Mironeasa and Gabriela [40]. The amount of minerals (ash) in TP was more than 10 times higher than in wheat flour (5.46 vs. 0.51 g/100 g), and in the crackers increased from 0.69 g/100 g (control) to a maximum of 1.22 g/100 g (10% TP). A high ash amount in bakery products (soda crackers, biscuits, and cakes) with different TP enrichment was also described by Alazb et al. [17], Isik and Topkaya [14], and Sogi et al. [41].

The lipid content in wheat flour was around 15 times lower compared to tomato pomace, where the lipids come from the seeds. Mironeasa and Gabriela [40] found 19.15 g/100 g lipids in flour made only from tomato seeds, i.e., concentrations slightly higher than those of our TP (that contained both peels and seeds). The cream crackers were prepared with margarine, an important source of lipids, and as a consequence, the amount of lipids in the control crackers was 11.39 g/100 g; nevertheless, in the enriched samples they increased significantly and progressively, reaching 13.04 in the 10% TP crackers. Accordingly, Chung [43] and Bhat and Ahsan [42] found a lipid increase in biscuits due to TP presence in their composition.

The protein content of tomato pomace was 20.73 g/100 g and in wheat flour 11.83 g/100 g; the high concentration in TP was due to the presence of tomato seeds, a good source of protein. Consequently, the protein level of control crackers differed statistically from that of the TP-enriched crackers. Increasing the content of protein by adding TP has been recorded in bread and muffins [10], biscuits [42], and soda crackers [14].

Tomato pomace is considered an excellent source of dietary fibre, and particularly of insoluble fibre [47]. In fact, the high dietary fibre content of TP is a leading reason for its use in enriching bakery products [15]. In our experiments, the soluble, insoluble, and total dietary fibre recorded in TP were 4.62, 32.00, and 36.62 g/100 g DM, respectively, i.e., several times more than in wheat flour (1.32, 2.51, and 3.83 g/100 g DM). The ANOVA (not presented) and the results in Figure 1 show that the amount of soluble, insoluble, and total dietary fibre in the enriched crackers was statistically different and superior to that of the control crackers. Other authors reached similar conclusions [10,14,17].

Starch is the main component of wheat flour and is far more abundant than in TP (86.20 vs. 57.42 g/100 g). Hence, in the TP-enriched crackers, a progressive decrease of carbohydrates at increasing tomato pomace levels was scored, as already noticed by Alazb et al. [17].

As mentioned above, tomato seeds (a component of tomato pomace) are rich in fatty acids [47], especially monounsaturated (MUFA) and polyunsaturated (PUFA) [11]. The fatty acids observed in our crackers are shown in Table 3.

The ANOVA (not presented) highlighted significant differences (*p* ≤ 0.05) among samples for all fatty acids. The palmitic, stearic, oleic, and linoleic acids in the control were 20.28, 4.16, 31.09, and 38.60 g/100 g oil, respectively, and increased progressively according to the TP enrichment of crackers, up to 21.35, 4.92, 33.10, and 41.74 g/100 g oil, respectively, in crackers with 10% TP. When grouping the fatty acids by their saturation level (Table 3), it was evident that all the crackers contained high amounts of MUFA (again, the role of the margarine should be remembered), and that the content increase of different groups of fatty acids was directly proportional to TP richness.

### 3.4. Carotenoids and Tocols

The HPLC analyses recorded the presence of lutein (1.26 mg/kg DM) and zeaxanthin (0.06 mg/kg DM) in the wheat flour, and of lutein (4.14 mg/kg DM), zeaxanthin (0.36 mg/kg DM), 15-cis-neurosporene (20.59 mg/kg DM), δ-carotene-isomer (10.00 mg/kg DM), and *trans*-lycopene (11.89 mg/kg DM) in the tomato pomace (Table 4). The total carotenoid content of TP (Figure 2) was >35 times greater than that of wheat flour (1.32 vs. 46.89 mg/kg DM). The significant differences among samples for total carotenoids detected by the ANOVA (not shown) were clearly reflected in the increasing concentrations found in the TP-enriched cream crackers (Figure 2), ranging from 6.83 mg/kg DM (4% TP) to 8.56 mg/kg (10% TP). The replacement of wheat flour with tomato pomace significantly increased the lycopene content of bread and muffin [10].

A more varied situation was observed for the tocols. In fact, the refined wheat flour contained only a limited quantity of β-tocotrienol (13.00 mg/kg DM), while the TP was rich in α- β-, γ-, and δ-tocopherols (55.30, 3.24, 116.65, and 3.47 mg/kg, respectively). Hence, the α-tocotrienols and δ-tocopherol found in the control crackers, as well as relevant quantities of the other tocopherols and tocotrienols, were due to the presence of margarine among the ingredients. Accordingly, the ANOVA demonstrated the existence of significant differences for total tocols contents between the control (44.38 mg/kg DM) and the four TP-enriched cream crackers (65.32–70.44 mg/kg DM), but not among the TP-enriched samples.

### 3.5. Phenolic Compounds

The refined wheat flour contained no free phenolics and only three insoluble bound phenolic acids (coumaric, ferulic, and ferulic derivative), while the TB contained several free and bound phenolic acids and flavonoids (Table 5). As for tocols, the phenolic composition of the cream crackers was influenced by the presence of the margarine. For example, a free phenolic acid retrieved in the control and enriched cream biscuits (4-hydroxybenzoic acid derivative), not found in either the wheat flour or the TP, may stem from the margarine. In fact, the main ingredient of the margarine we used was oil from palm oil, whose fruits are very rich in 4-hydroxybenzoic acid [48], and the high temperatures during oil processing may induce a transformation towards a less polar compound. Additionally, the high and increasing concentrations of free gallic acid in the TP-enriched samples may be due to its release from different TP compounds during the long yeast fermentation step [49,50]. A less likely explanation could be the heat effect during baking, because in a yeast-less cake enriched with grape pomace, the baking step did not elicit an increase in gallic acid [39].

**Figure 3:**
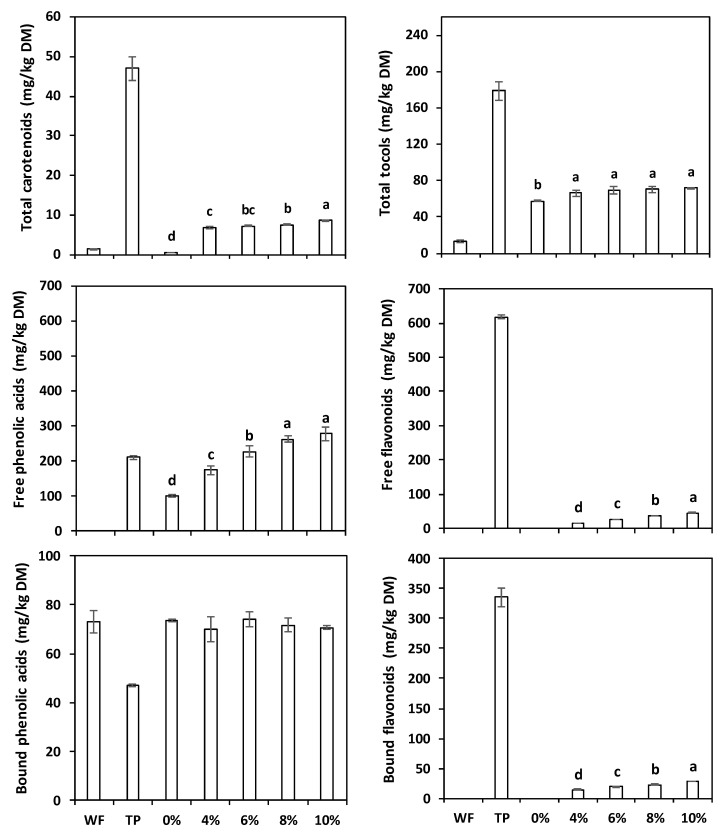
Total carotenoids, total tocols, soluble free phenolic acids and flavonoids, insoluble bound phenolic acids and flavonoid contents of bread wheat flour (WF), tomato pomace (TP), and crackers prepared with increasing TP percentages. The error bars represent the standard deviation; different letters indicate significant differences (*p* < 0.05) among cream crackers.

While the bread wheat flour contained only bound phenolic acids, the TP showed high concentrations of both free and bound phenolics (Figure 3); furthermore, TP was very rich in flavonoids. Overall, the tomato pomace polyphenol content was more than 10 times (1211.4 vs. 73.2 mg/kg DM) that of the refined wheat flour; additionally, most of its compounds were of the free form (828.34 vs. 383.08 mg/kg DM), which has a better nutritional significance for superior bioaccessibility and bioavailability [26,51]; hence, adding TP, rich in free phenolics, will significantly improve the polyphenol composition of the cream crackers. Alazb et al. [17], using a different extraction protocol with alcohol, recovered 653.17 μg/g DM of phenolic compounds, mainly constituted (45%) by kaempferol.

The increasing TP concentrations in the enriched cream crackers led to a progressive augmentation of several soluble free phenolic acids (p-coumaric, p-coumaric der, ferulic, gallic) and flavonoids (naringenin, naringenin der, quercitin der, rutin), as well as of some insoluble bound phenolic acid (caffeic) and flavonoids (naringenin, naringenin der, quercitin der). Overall, the addition of 10% TP increased the free phenolics and bound phenolics contents by 322% and 133%, respectively, in comparison to the control, while the free-to-bound phenolics ratio jumped from 1.36 (control) to 3.28 (10% TP), significantly improving the nutritional characteristics of the cream crackers.

Isik and Topkaya [14] partially replaced wheat flour with 4%, 8%, and 12% dried tomato pomace meal in the preparation of crackers and noticed a concomitant total polyphenol content (TPC) increase from 52.52 to 127.58 mg GAE/100 g. Yagci et al. [52] concluded that increasing the levels of tomato pomace powder in extruded snacks significantly increased the content of individual free phenolics, including gallic acid, protocatechuic acid, 2,5-dihydroxybenzoic acid, chlorogenic acid, rutin, and quercetin.

### 3.6. Antioxidant Capacity

**Figure 4:**
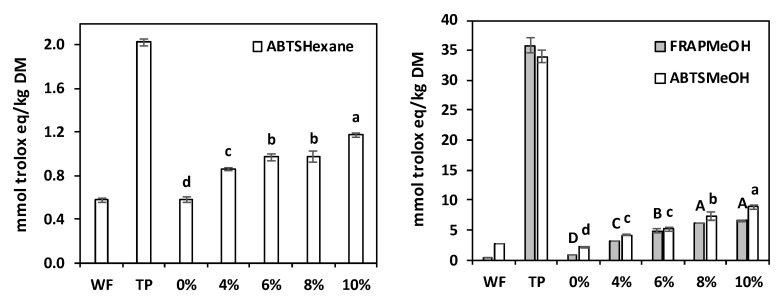
Antioxidant capacity (FRAP and ABTS tests) of the lipophilic extracts (in hexane) and hydrophilic extracts (in 80% methanol) of bread wheat flour (WF), tomato pomace (TP), and crackers prepared with increasing TP percentages. The error bars represent the standard deviation; different letters indicate significant differences (*p* ≤ 0.05) among cream crackers.

The antioxidant capacities of the lipophilic and hydrophilic extracts are depicted in Figure 4. The lipophilic extracts (carotenoids and tocols) showed limited antioxidant capacity in comparison to the hydrophilic extracts, possibly because of the much higher concentrations of phenolic compounds. The TP always displayed far higher values than the refined wheat flour. Accordingly, the TP-enriched cream cookies exhibited significantly increased antioxidant capacities when their TP content was augmented.

### 3.7. Sensory Analysis

**Figure 5:**
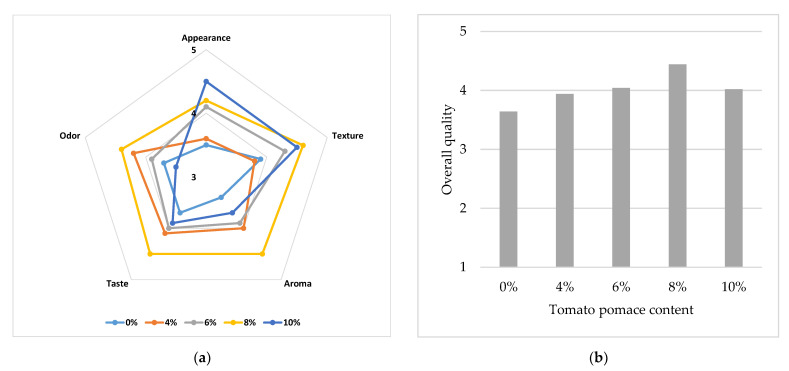
Sensory analysis results (**a**) and overall quality (**b**) of crackers prepared with increasing TP percentages.

The sensory analysis showed that the cream crackers with TP had good acceptability, insofar that those enriched with 10% TP displayed the best score for appearance, while those with 8% TP were the best for texture, aroma, taste, and odour (Figure 5a), as well as for overall acceptability, with a score of 4.4/5.0 points (Figure 5b). Mehta et al. [10] observed that adding TP in bakery products (bread and muffins) made their colour more appealing.

The optimal 8% TP percentage for most of the parameters as well as for overall quality is consistent with Isik and Topkaya [14], who concluded that the addition of 12% or more TP in the production of soda crackers is not recommended, and with Bhat and Ahsan [42], who observed that biscuits with 5% TP received the best assessment. Similarly, Alazb et al. [17] did not record significant differences between a control cake and samples enriched with 2.5, 5, and 7.5% tomato pomace. The level of TP addition may be lower in high-rising leavened products such as bread. Majzoobi et al. [37] reported that bread with 5% and 7% TP was worse than the control sample, while Nour et al. [45] reported that bread with 6% TP was better than bread with 10% TP.

## 4. Conclusions

Tomato pomace is a by-product with excellent nutritional composition (high ash, fat, protein, and dietary fibre contents). Cream crackers enriched with different TP amounts (4%, 6%, 8%, and 10%) showed better physical and nutritional characteristics than the control, and the improvements were directly correlated to the TP content. The sensory analysis showed that crackers with 8% TP had the best acceptance among panellists. These results can help to promote the use of different nutrient-rich by-products from the food industry in the production of new functional foods.

## Figures and Tables

**Table 1:** Pasting properties (mean ± standard deviation) of the five blends used for the preparation of crackers with increasing quantities of tomato pomace (TP).

	0% TP	4% TP	6% TP	8% TP	10% TP
Peak viscosity (BU)	895.0 ± 12.7 ^d^	898.5 ± 2.1 ^d^	946.0 ± 5.7 ^c^	971.0 ± 5.7 ^b^	1024.0 ± 5.7 ^a^
Breakdown (BU)	297.0 ± 4.2 ^d^	308.0 ± 2.8 ^d^	345.0 ± 2.8 ^c^	390.0 ± 14.1 ^b^	422.5 ± 10.6 ^a^
Final viscosity (BU)	1042.5 ± 14.9 ^a^	1047.5 ± 14.9 ^bc^	1058.0 ± 4.2 ^bc^	1068.0 ± 2.8 ^b^	1147.0 ± 10.0 ^a^
Pasting temperature (°C)	60.7 ± 0.6 ^b^	61.3 ± 0.3 ^ab^	61.3 ± 0.1 ^ab^	61.5 ± 0.5 ^ab^	61.8 ± 0.2 ^a^

Values in the same row with different letters are significantly different (*p* < 0.05) following Fisher’s LSD test. BU: Brabender Units.

**Table 2:** Colour, physical, and textural characteristics (mean ± standard deviation) of crackers with increasing quantities of tomato pomace (TP).

	0% TP	4% TP	6% TP	8% TP	10% TP
Colour					
			Dough		
L*	68.5 ± 2.5 ^a^	68.1 ± 2.5 ^a^	65.6 ± 2.2 ^b^	62.8 ± 2.8 ^ab^	58.1 ± 1.3 ^c^
a*	−0.4 ± 0.04 ^e^	5.4 ± 0.9 ^d^	8.1 ± 0.3 ^c^	9.7 ± 0.8 ^b^	10.9 ± 0.9 ^a^
b*	19.7 ± 0.3 ^d^	25.7 ± 0.4 ^c^	27.8 ± 0.4 ^b^	28.3 ± 0.1 ^ab^	28.4 ± 0.5 ^a^
			Crackers		
L*	69.7 ± 0.8 ^a^	66.1 ± 1.2 ^b^	61.9 ± 0.7 ^c^	61.0 ± 1.6 ^c^	58.4 ± 2.3 ^d^
a*	−0.7 ± 0.03 ^a^	3.8 ± 0.7 ^a^	6.8 ± 1.2 ^a^	7.3 ± 0.3 ^b^	7.6 ± 1.6 ^c^
b*	16.9 ± 0.2 ^a^	23.9 ± 0.6 ^ab^	25.3 ± 2.3 ^bc^	26.9 ± 1.1 ^c^	27.5 ± 1.2 ^d^
Physical and textural characteristics					
Width (mm)	35.8 ± 0.0 ^a^	35.1 ± 3.0 ^a^	34.9 ± 2.0 ^a^	34.3 ± 0.7 ^a^	34.5 ± 1.8 ^a^
Thickness (mm)	8.4 ± 0.9 ^a^	7.8 ± 0.0 ^ab^	7.6 ± 0.4 ^ab^	6.7 ± 0.2 ^bc^	6.1 ± 0.1 ^c^
Spread factor	4.3 ± 0.5 ^b^	4.5 ± 0.4 ^b^	4.6 ± 0.5 ^b^	5.2 ± 0.3 ^ab^	5.7 ± 0.2 ^a^
Volume (cm^3^)	7.1 ± 0.1 ^a^	4.9 ± 0.1 ^b^	4.6 ± 0.3 ^b^	3.7 ± 0.1 ^c^	3.5 ± 0.0 ^c^
Specific volume (cm^3^/g)	1.8 ± 0.1 ^a^	1.7 ± 0.0 ^ab^	1.5 ± 0.01 ^bc^	1.5 ± 0.1 ^c^	1.4 ± 0.1 ^c^
Hardness (N)	65.4 ± 9.5 ^a^	42.7 ± 5.0 ^b^	36.4 ± 3.5 ^bc^	30.0 ± 2.7 ^cd^	26.3 ± 4.4 ^d^
Snapping force (N)	4.8 ± 2.6 ^d^	8.0 ± 1.9 ^c^	10.4 ± 1.7 ^bc^	12.0 ± 1.9 ^ab^	13.8 ± 3.0 ^a^

Values in the same row with different letters are significantly different (*p* < 0.05) following Fisher’s LSD test.

**Table 3:** Fatty acids (%) of crackers with different quantities of tomato pomace (TP).

	0% TP	4% TP	6% TP	8% TP	10% TP
Palmitic	20.28 ± 0.01 ^d^	20.54 ± 0.01 ^c^	20.58 ± 0.07 ^c^	20.88 ± 0.01 ^b^	21.35 ± 0.01 ^a^
Stearic	4.16 ± 0.00 ^e^	4.18 ± 0.00 ^d^	4.54 ± 0.01 ^c^	4.77 ± 0.00 ^b^	4.92 ± 0.02 ^a^
Oleic	31.09 ± 0.01 ^d^	32.04 ± 0.01 ^c^	32.06 ± 0.11 ^c^	32.19 ± 0.01 ^b^	33.10 ± 0.01 ^a^
Linoleic	38.60 ± 0.14 ^d^	39.24 ± 0.02 ^c^	39.31 ± 0.01 ^c^	40.66 ± 0.01 ^b^	41.74 ± 0.01 ^a^
MUFA	33.52 ± 0.01 ^e^	33.99 ± 0.01 ^d^	34.28 ± 0.02 ^c^	33.92 ± 0.11 ^b^	33.75 ± 0.01 ^a^
PUFA	40.64 ± 0.16 ^d^	40.41 ± 0.01 ^c^	40.21 ± 0.01 ^c^	40.78 ± 0.02 ^b^	40.94 ± 0.02 ^a^
SFA	25.83 ± 0.01 ^d^	25.62 ± 0.01 ^c^	25.53 ± 0.01 ^b^	25.52 ± 0.09 ^a^	25.35 ± 0.01 ^a^

Values in the same row with different letters are significantly different (*p* < 0.05) following Fisher’s LSD test. MUFA, monounsaturated fatty acids; PUFA, polyunsaturated fatty acids; SFA, saturated fatty acids.

**Table 4:** Carotenoids and tocols contents (mg/kg DM; mean ± standard deviation) of wheat flour (WF), tomato pomace (TP), and cream crackers with increasing TP percentages. Different letters indicate significant differences (*p* < 0.05) among cream crackers along a row.

	WF	TP	0%	4%	6%	8%	10%
Carotenoids							
Lutein	1.26 ± 0.06	4.14 ± 0.24	0.53 ± 0.03	0.64 ± 0.07	0.59 ± 0.05	0.57 ± 0.02	0.59 ± 0.01
Zeaxanthin	0.06 ± 0.00	0.36 ± 0.03	0.02 ^c^ ± 0.00	0.04 ^b^ ± 0.01	0.04 ^b^ ± 0.01	0.04 ^ab^ ± 0.00	0.05 ^a^ ± 0.00
15-*cis*-neurosporene	nd	20.59 ± 1.43	nd ^d^	2.24 ^c^ ± 0.22	2.46 ^bc^ ± 0.19	2.77 ^b^ ± 0.11	3.47 ^a^ ± 0.19
δ-carotene-isomer	nd	10.00 ± 1.04	nd	1.56 ± 0.03	1.61 ± 0.02	1.63 ± 0.07	1.69 ± 0.02
*trans*-lycopene	nd	11.89 ± 0.75	nd ^c^	2.35 ^b^ ± 0.09	2.49 ^b^ ± 0.04	2.53 ^b^ ± 0.09	2.76 ^a^ ± 0.00
Tocols							
α-tocopherol	nd	55.30 ± 5.19	18.52 ^c^ ± 0.45	21.40 ^b^ ± 1.27	23.65 ^a^ ± 0.92	24.10 ^a^ ± 0.28	25.46 ^a^ ± 0.17
α-tocotrienol	nd	nd	7.76 ± 0.62	9.10 ± 0.71	8.83 ± 0.91	8.64 ± 0.91	8.81 ± 0.04
β-tocopherol	nd	3.24 ± 0.38	2.53 ± 0.05	2.05 ± 0.34	3.03 ± 0.90	2.25 ± 0.81	2.24 ± 0.18
β-tocotrienol	13.00 ± 0.85	nd	13.21 ± 0.29	14.51 ± 1.24	14.15 ± 1.43	14.47 ± 0.92	14.36 ± 0.11
γ-tocopherol	nd	116.65 ± 4.03	nd ^d^	2.17 ^c^ ± 0.37	2.80 ^b^ ± 0.25	3.94 ^a^ ± 0.03	4.55 ^a^ ± 0.01
δ-tocopherol	nd	nd	15.57 ± 0.24	16.09 ± 0.41	15.43 ± 1.30	15.20 ± 1.18	15.02 ± 0.14
δ-tocotrienol	nd	3.47 ± 0.14	nd	0.90 ± 0.09	1.55 ± 0.32	1.60 ± 0.10	1.19 ± 0.30

nd: not detected, i.e., inferior to the detection limit.

**Table 5:** Phenolics content (mean ± standard deviation) of wheat flour (WF), tomato pomace (TP), and cream crackers with increasing TP percentages. Different letters indicate significant differences (*p* ≤ 0.05) among cream crackers along a row.

	WF	TP	0%	4%	6%	8%	10%
				**Soluble free**			
Phenolic acids							
4OH-benzoic acid der			87.60 ± 4.23	94.78 ± 8.48	92.90 ± 9.21	91.06 ± 3.40	92.21 ± 8.44
Coumaric acid		6.84 ± 0.10		0.40 ^d^ ± 0.02	0.57 ^c^ ± 0.01	0.74 ^b^ ± 0.02	0.92 ^a^ ± 0.01
Coumaric acid der		49.97 ± 2.15		3.09 ^d^ ± 0.11	4.27 ^c^ ± 0.08	5.52 ^b^ ± 0.10	6.35 ^a^ ± 0.01
Ferulic acid		28.22 ± 1.13	2.55 ^c^ ± 0.03	4.30 ^b^ ± 0.04	4.28 ^b^ ± 0.00	4.44 ^b^ ± 0.12	5.13 ^a^ ± 0.08
Gallic acid		81.98 ± 2.98	9.93 ^d^ ± 0.17	70.99 ^c^ ± 2.80	124.81 ^b^ ± 7.23	160.65 ^a^ ± 4.49	173.12 ^a^ ± 10.93
Syringic acid		42.53 ± 0.80					
Flavonoids							
Naringenin		39.02 ± 0.14		2.96 ^c^ ± 0.24	5.20 ^b^ ± 0.10	6.98 ^a^ ± 0.31	7.69 ^a^ ± 0.35
Naringenin der		33.78 ± 0.27		0.73 ^d^ ± 0.01	1.28 ^c^ ± 0.04	1.84 ^b^ ± 0.13	2.39 ^a^ ± 0.01
Quercitin der		56.74 ± 0.24		1.86 ^d^ ± 0.02	3.23 ^c^ ± 0.11	4.15 ^b^ ± 0.04	5.00 ^a^ ± 0.20
Rutin		489.26 ± 6.50		9.81 ^d^ ± 0.03	17.26 ^c^ ± 0.18	23.76 ^b^ ± 0.55	30.20 ^a^ ± 1.17
				**Insoluble bound**			
Phenolic acids							
Caffeic acid		29.75 ± 0.18					
Coumaric acid	0.16 ± 0.01	5.54 ± 0.65	0.16 ^c^ ± 0.01	0.36 ^b^ ± 0.03	0.44 ^b^ ± 0.04	0.53 ^a^ ± 0.03	0.58 ^a^ ± 0.05
Coumaric acid der		0.26 ± 0.01					
Ferulic acid	67.28 ± 4.21	11.47 ± 0.92	67.35 ± 0.44	63.93 ± 4.79	67.65 ± 3.09	65.33 ± 2.86	64.25 ± 0.51
Ferulic acid der	5.79 ± 0.38		6.16 ± 0.02	5.88 ± 0.22	6.09 ± 0.03	5.91 ± 0.03	5.83 ± 0.41
Flavonoids							
Naringenin		172.46 ± 11.81		3.92 ^c^ ± 0.31	6.05 ^b^ ± 0.50	6.86 ^ab^ ± 0.67	8.10 ^a^ ± 0.41
Naringenin der		65.47 ± 2.29		4.23 ^b^ ± 0.07	5.50 ^a^ ± 0.53	5.47 ^a^ ± 0.06	6.37 ^a^ ± 0.40
Quercitin der		98.14 ± 6.23		5.77 ^c^ ± 0.57	7.46 ^c^ ± 0.59	10.28 ^b^ ± 1.07	13.24 ^a^ ± 0.59

## Data Availability

Data are contained within the article.
